# Trained immunity in chronic rhinosinusitis: epigenetic reprogramming of innate immune memory as a driver of mucosal inflammation and recurrence

**DOI:** 10.3389/fimmu.2026.1822395

**Published:** 2026-07-15

**Authors:** Guan-Jiang Huang, Ling-Juan Li, Pei-Shan Li, Zhi-Jun Fan, Biao-Qing Lu

**Affiliations:** 1 Department of Otorhinolaryngology Head and Neck Surgery, Zhongshan Hospital of Traditional Chinese Medicine, Affiliated to Guangzhou University of Chinese Medicine, Zhongshan, Guangdong, China; 2 The Tenth Clinical Medical College of Guangzhou University of Chinese Medicine, Zhongshan, Guangdong, China

**Keywords:** chronic rhinosinusitis, innate immune memory, mucosal inflammation, nasal polyp, trained immunity

## Abstract

Chronic rhinosinusitis (CRS) is a highly prevalent and debilitating inflammatory condition of the upper airway, affecting 5-28% of the global population and imposing a substantial socioeconomic burden. Despite major advances in endoscopic sinus surgery, pharmacological management, and targeted biologic therapies, long-term disease recurrence following treatment remains an unresolved clinical challenge. Current pathophysiological frameworks centered on adaptive type 2 immunity, eosinophilic inflammation, and pathogen persistence fail to fully account for the chronification and therapy resistance of CRS. Emerging evidence positions trained immunity (the epigenetic and metabolic reprogramming of innate immune cells enabling non-antigen-specific functional memory) as a fundamental and previously underappreciated mechanism driving CRS recurrence. Persistent sinonasal microbial colonizers, including *Staphylococcus aureus* biofilms and fungal components, along with viral pathogens and dysbiotic microbiome communities, function as potent epigenetic training stimuli that reprogram sinonasal macrophages, group 2 innate lymphoid cells (ILC2s), and epithelial progenitor cells. The recent identification of a TLR4+ trained ILC2 subset in nasal polyp tissue, sustained by AP-1-driven chromatin remodeling at the *Tlr4* locus, exemplifies the cellular specificity of this phenomenon. Concurrently, nasal basal stem cells acquire heritable pro-inflammatory chromatin states following type 2 cytokine exposure, encoding an epithelial inflammatory memory that perpetuates mucosal dysfunction independent of ongoing stimulation. This review systematically examines the microbial triggers, epigenetic mechanisms, key cellular mediators, and therapeutic implications of trained immunity in CRS, proposing a new framework for disease-modifying strategies targeting the sinonasal epigenetic inflammatory landscape.

## 1 Introduction

Chronic rhinosinusitis (CRS) is defined as persistent inflammatory disease of the nasal cavity and paranasal sinuses lasting more than 12 weeks, manifesting clinically as nasal obstruction, purulent rhinorrhea, facial pressure or pain, and olfactory dysfunction (1, 2). Affecting an estimated 5-28% of the global population and necessitating over one million surgical procedures annually, CRS imposes a profound socioeconomic burden, with annual healthcare costs exceeding billions of dollars in Western countries (2–4). Conventionally stratified into CRS with nasal polyps (CRSwNP) and without (CRSsNP), and further classified by immunological endotype into type 2 and non-type 2 inflammatory subtypes, CRS is now recognized as a highly heterogeneous disorder demanding precision therapeutic approaches (5–9). The contemporary management landscape for CRS has undergone substantial transformation. Endoscopic sinus surgery (ESS) combined with topical corticosteroid therapy remains the standard of care, while biologic agents, most notably dupilumab (anti-IL-4Rα), targeting the IL-4/IL-13 signaling axis, have dramatically improved outcomes in severe type 2 CRSwNP (1, 10–13). Yet a clinically disquieting reality persists: longitudinal follow-up studies document disease recurrence in the majority of ESS-treated patients within years of surgery, and symptoms invariably worsen following biologic discontinuation (10, 14). This recalcitrant trajectory suggests that current therapeutic strategies, even those targeting canonical type 2 immune effectors, fail to fundamentally alter the underlying mucosal inflammatory program (15).

It remains unclear why inflammation persists and recurs despite pathogen clearance and potent immunotherapy. Addressing this demands mechanistic insights beyond conventional viewpoints. We propose that trained immunity provides this answer. First formally articulated by Netea, Quintin, and van der Meer in 2011 (16), trained immunity denotes the capacity of innate immune cells to exhibit enhanced and sustained responsiveness upon secondary stimulation through non-antigen-specific, epigenetically mediated functional memory (17, 18). Unlike classical adaptive immune memory, trained immunity operates through histone modifications, alterations in DNA methylation, and metabolic reprogramming, collectively enabling macrophages, group 2 innate lymphoid cells (ILC2s), and epithelial progenitor cells to “remember” prior inflammatory encounters and mount amplified responses to subsequent stimuli, including benign or low-grade triggers (19–21). The past five years have witnessed an exponential growth in trained immunity research, with a rapidly expanding body of review literature consolidating mechanistic, cellular, and translational dimensions of this field (22, 23). In the context of type 2 allergic inflammation specifically, Schiller et al. recently synthesized evidence demonstrating that trained immunity in dendritic cells and macrophages actively promotes Th2-skewed responses and may modulate the efficacy of allergen immunotherapy (22).

The CRS sinonasal mucosa represents a uniquely permissive environment for the induction of maladaptive trained immunity. Chronically colonized by pathogens including *Staphylococcus aureus*, intermittently exposed to fungal antigens and respiratory viruses, and subject to dysbiotic microbiome perturbation, the sinonasal mucosa is persistently bombarded with the very microbial signals that most potently induce trained immunity in macrophages and innate lymphoid cells (15, 24, 25). This review systematically explores trained immunity as a unifying pathophysiological framework for CRS chronification. We elucidate the microbial and environmental triggers of sinonasal trained immunity, detail the epigenetic and metabolic reprogramming of key mucosal populations (macrophages, ILC2s, and epithelial progenitors), and discuss the therapeutic implications of targeting the trained immune state in CRS.

## 2 Conceptual foundations of trained immunity

### 2.1 Beyond classical immunological memory: the innate dimension

**Figure 1:**
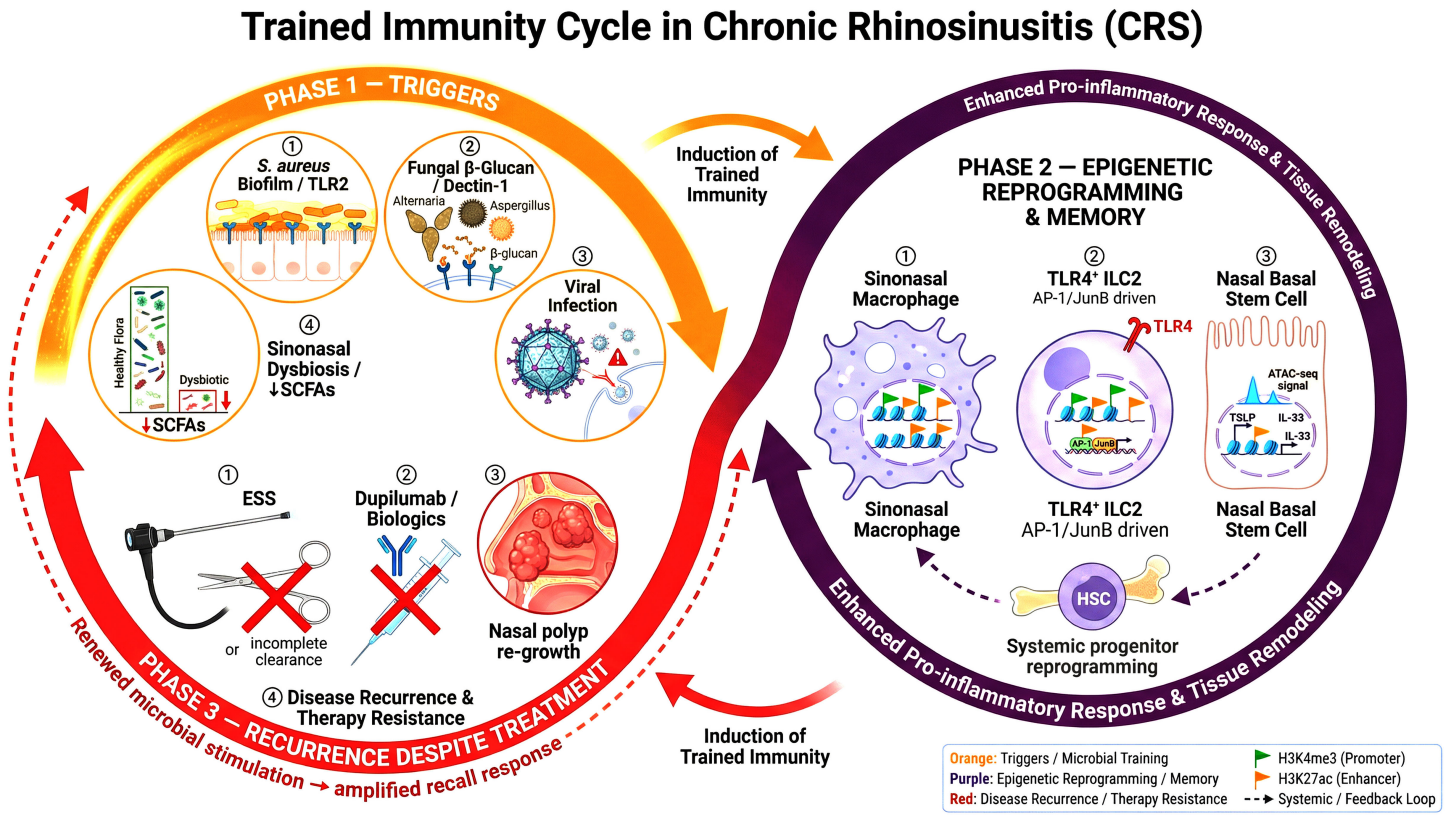
The trigger, memory, and recurrence cycle of trained immunity in chronic rhinosinusitis (CRS). Three interconnected phases perpetuate a self-reinforcing sinonasal inflammatory cycle. Phase 1 (Triggers, orange): Four stimuli induce trained immunity, namely S. aureus biofilm via TLR2, fungal β-glucan engaging Dectin-1, viral infection, and sinonasal dysbiosis with depletion of SCFA-producing commensals. Phase 2 (Epigenetic Reprogramming and Memory, purple): Microbial stimuli deposit permissive histone marks (H3K4me3 and H3K27ac) across three cellular populations, including sinonasal macrophages, TLR4^+^ ILC2s (AP-1/JunB-driven), and nasal basal stem cells (open chromatin at TSLP and IL-33 loci). Systemic inflammatory mediators concurrently reprogram bone marrow hematopoietic stem cells (HSCs), sustaining a pre-activated progenitor supply to the mucosa. Phase 3 (Recurrence Despite Treatment, red): Epigenetically imprinted cells persist despite endoscopic sinus surgery and biologics (e.g., dupilumab), mounting amplified recall responses upon re-stimulation and driving nasal polyp regrowth. A feedback loop from Phase 3 to Phase 1 illustrates how post-surgical mucosal re-colonization perpetuates the cycle. AP-1, Activator Protein 1; CRS, Chronic Rhinosinusitis; Dectin-1, Dendritic Cell-Associated C-type Lectin-1; H3K4me3, Histone H3 Lysine 4 Trimethylation; H3K27ac, Histone H3 Lysine 27 Acetylation; HSC, Hematopoietic Stem Cell; ILC2, Group 2 Innate Lymphoid Cell; IL-33, Interleukin-33; JunB, Jun B Proto-oncogene; S. aureus, Staphylococcus aureus; SCFA, Short-Chain Fatty Acid; TLR2, Toll-Like Receptor 2; TLR4, Toll-Like Receptor 4; TSLP, Thymic Stromal Lymphopoietin.

Traditionally defined as the capacity to mount a rapid and robust response upon re-exposure to a stimulus, immunological memory has long been considered the exclusive hallmark of adaptive immunity, conferred by long-lived, antigen-specific T and B lymphocytes (16). This paradigm was fundamentally challenged by accumulating evidence from diverse biological systems, including the non-specific cross-protection afforded by Bacillus Calmette–Guérin (BCG) vaccination in immunodeficient mice lacking T and B cells, and the capacity of plants and invertebrates to “remember” prior infectious encounters despite lacking adaptive immune machinery (18, 26, 27). These observations led Netea and colleagues to formally define trained immunity as an enhanced immune response of innate immune cells to rechallenges, resulting from functional reprogramming mediated by epigenetic and metabolic mechanisms rather than somatic recombination (16, 17, 28). The trigger, memory, and recurrence cycle of trained immunity in CRS were displayed in **Figure 1**.

The mechanistic architecture of trained immunity is built upon two deeply interconnected and mutually reinforcing pillars, namely epigenetic reprogramming and metabolic rewiring, with bidirectional crosstalk between these axes being indispensable for both the induction and long-term maintenance of the trained innate immune state (19, 29–31). The epigenetic pillar of trained immunity involves the deposition of permissive histone marks (most prominently, trimethylation of histone H3 at lysine 4 [H3K4me3] at gene promoters and acetylation of histone H3 at lysine 27 [H3K27ac] at active enhancers) at loci encoding pro-inflammatory mediators, thereby enabling their more rapid and robust transcriptional activation upon secondary stimulation (19). These chromatin modifications persist beyond the resolution of the initial stimulus, creating epigenetically “poised” regulatory elements (19, 29, 31). The metabolic pillar involves a functional shift toward aerobic glycolysis (the Warburg effect), fumarate accumulation through glutamine anaplerosis of the tricarboxylic acid (TCA) cycle, and activation of the mTOR/HIF-1α signaling axis. Crucially, this metabolic rewiring not only fulfills biosynthetic demands but also directly regulates epigenetic enzyme activity by altering the availability of key co-substrates, such as acetyl-CoA and succinate (32–34).

The direct relevance of this metabolic pillar to CRS pathophysiology is compellingly supported by convergent multi-compartment evidence. Haimerl et al. documented constitutively elevated acylcarnitines in monocyte-derived macrophages, nasal lining fluid, sputum, and plasma of N-ERD patients, alongside genome-wide differential methylation at CPT1A, CPT1B, and ACACA loci encoding key fatty acid oxidation enzymes, thereby establishing that pathological macrophage trained immunity in CRS is inseparably metabolic and epigenetic in character (35). Current evidence further positions the mTOR/HIF-1α/glycolytic axis as the central metabolic-epigenetic interface driving trained immunity induction in CRS mucosal macrophages (29, 32).

### 2.2 Central vs peripheral trained immunity: implications for CRS

A critical conceptual distinction concerns the anatomical site of trained immune reprogramming. Distinct from central mechanisms, peripheral trained immunity entails the localized epigenetic and functional reprogramming of tissue-resident innate immune populations (e.g., mucosal macrophages and ILC2s), which is triggered *in situ* by local stimuli (17). This form of trained immunity is most immediately relevant to the sinonasal mucosa, where persistent microbial colonization continuously shapes the epigenetic landscape of resident immune cells (15). Central trained immunity, in contrast, refers to the epigenetic reprogramming of hematopoietic stem cells (HSCs) and myeloid progenitor cells in the bone marrow, resulting in the sustained generation of functionally pre-activated monocyte progeny (21, 30, 36). Seminal studies by Kaufmann et al. and Mitroulis et al. demonstrated that BCG vaccination and β-glucan exposure, respectively, induce lasting epigenetic changes in HSCs, generating monocyte offspring with constitutively enhanced pro-inflammatory capacity (36, 37). In the CRS context, this central mechanism elegantly explains why trained immunity can persist for months and why ESS and biologic therapy fail to permanently normalize mucosal immune reactivity (15, 38).

### 2.3 The pathological face of trained immunity

While trained immunity confers adaptive advantages in host defense against reinfection, its aberrant or excessive induction drives chronic inflammatory pathology (20, 38). Pathological trained immunity has been documented across a spectrum of chronic inflammatory and autoimmune diseases, including systemic lupus erythematosus, lupus nephritis, gout, stroke, and systemic sclerosis (38–40). Under these conditions, innate immune cells adopt a hyperreactive phenotype that exacerbates tissue damage upon exposure to otherwise benign stimuli. This mechanism is central to CRS, where routine encounters with airborne microbes and aeroallergens trigger disproportionate inflammatory cascades and sustain polyp development (15, 24).

## 3 Microbial and environmental triggers of trained immunity in the sinonasal mucosa

**Figure 2:**
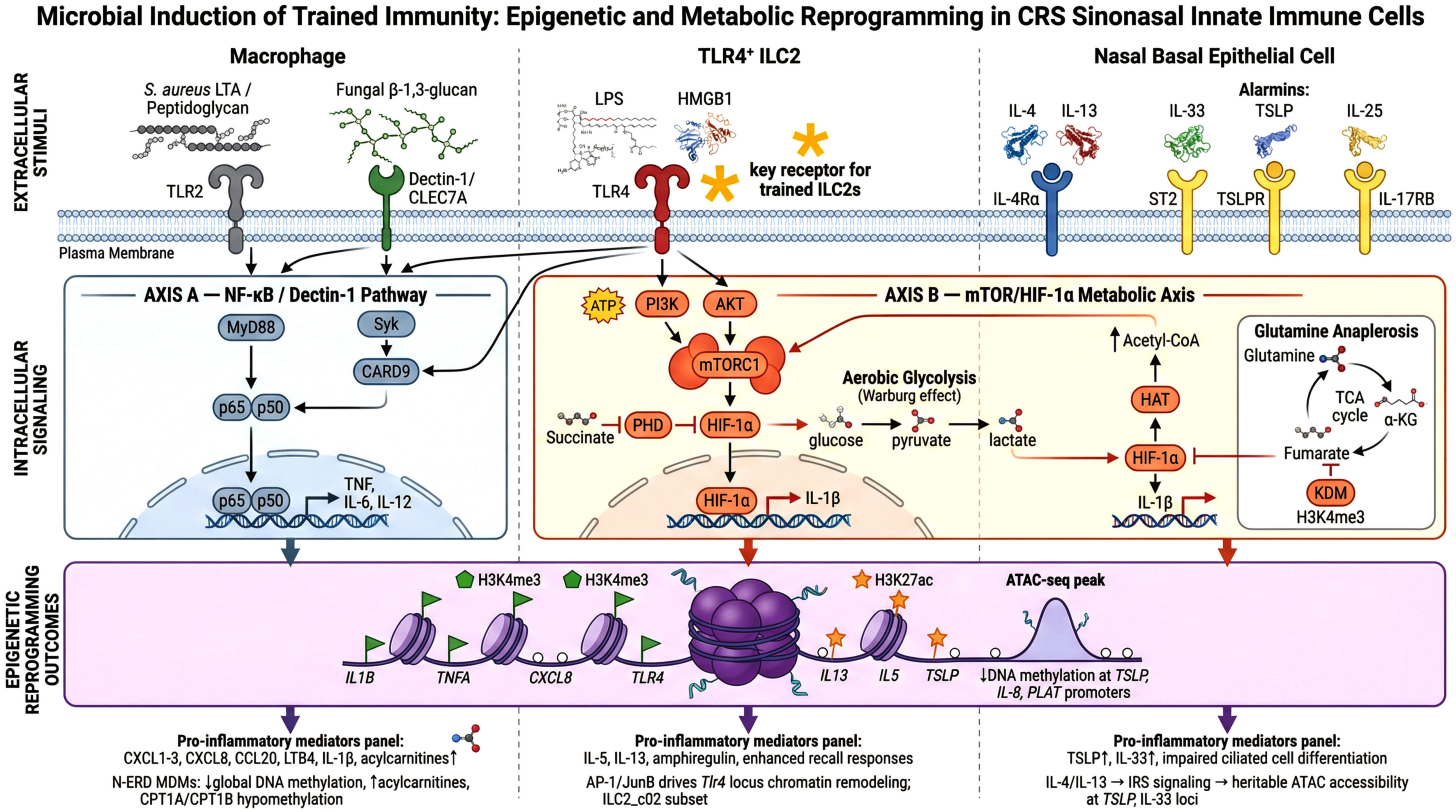
Molecular signaling pathways linking sinonasal microbial exposure to epigenetic reprogramming in CRS innate immune and epithelial cells. Three parallel cell columns (sinonasal macrophage, TLR4^+^ ILC2, and nasal basal epithelial cell) share a common stimulus layer (top) and converge on epigenetic outcomes (bottom). Top (Extracellular Stimuli): S. aureus LTA/peptidoglycan engages TLR2, fungal β-1,3-glucan engages Dectin-1/CLEC7A, and LPS/HMGB1 engages TLR4 (key trained ILC2 receptor). IL-4, IL-13, and alarmins (IL-33, TSLP, IL-25) activate basal epithelial receptors. Middle (Intracellular Signaling): Axis A (NF-κB/Dectin-1) drives MyD88 and Syk/CARD9 cascades. Axis B (mTOR/HIF-1α) couples PI3K/AKT to mTORC1 activation and aerobic glycolysis (Warburg effect). Succinate stabilizes HIF-1α, fumarate inhibits KDM, and elevated acetyl-CoA fuels HAT-mediated histone modification. Bottom (Epigenetic Outcomes): H3K4me3 marks at IL1B, TNFA, CXCL8, and TLR4, H3K27ac at IL13, IL5, and TSLP, and reduced DNA methylation at TSLP, IL-8, and PLAT promoters, producing cell-type-specific pro-inflammatory mediator outputs. Acetyl-CoA, acetyl coenzyme A; AKT, protein kinase B (AKT serine/threonine kinase); CARD9, caspase recruitment domain family member 9; CLEC7A (Dectin-1), C-type lectin domain containing 7A; CRS, chronic rhinosinusitis; CXCL8 (IL-8), C-X-C motif chemokine ligand 8; H3K4me3, histone H3 lysine 4 trimethylation; H3K27ac, histone H3 lysine 27 acetylation; HAT, histone acetyltransferase; HIF-1α, hypoxia-inducible factor 1-alpha; HMGB1, high mobility group box 1; IL, interleukin; IL1B, interleukin-1 beta (gene locus); IL5, interleukin-5 (gene locus); IL13, interleukin-13 (gene locus); ILC2, group 2 innate lymphoid cell; KDM, lysine demethylase; LPS, lipopolysaccharide; LTA, lipoteichoic acid; mTOR, mechanistic target of rapamycin; mTORC1, mechanistic target of rapamycin complex 1; MyD88, myeloid differentiation primary response 88; NF-κB, nuclear factor kappa-light-chain-enhancer of activated B cells; PI3K, phosphoinositide 3-kinase; PLAT, plasminogen activator tissue type (gene locus); Syk, spleen tyrosine kinase; TLR2, Toll-like receptor 2; TLR4, Toll-like receptor 4; TNFA, tumor necrosis factor alpha (gene locus); TSLP, thymic stromal lymphopoietin.

Molecular signaling pathways linking sinonasal microbial exposure to epigenetic reprogramming in CRS innate immune and epithelial cells were displayed in **Figure 2**.

### 3.1 *Staphylococcus aureus* and biofilm-mediated epigenetic imprinting

Among the diverse microbial inhabitants of the sinonasal mucosa, *Staphylococcus aureus* occupies a position of singular importance in CRS pathogenesis, particularly in severe and recalcitrant CRSwNP (25). S. *aureus* colonizes the nasal mucosa in a substantial proportion of CRSwNP patients and forms resilient biofilms that resist antibiotic eradication, provide a chronic source of inflammatory stimuli, and are associated with the most severe and treatment-resistant disease phenotypes (15, 25). Beyond its direct proinflammatory effects through TSLP and IL-33 induction in nasal epithelial cells, emerging evidence positions S. *aureus* as a potent inducer of trained immunity in sinonasal innate immune cells (25).

S. *aureus* expresses superantigens (SAgs, including staphylococcal enterotoxins A and B) that activate large fractions of T cells in a TCR Vβ-restricted manner, driving polyclonal type 2 cytokine secretion (41). Our group has demonstrated that superantigen-related expansion of TCR Vβ5.1+CD4+ T cells with Th2 phenotypes (expressing high levels of CRTH2, IL-17RB, and ST2) constitutes a defining immunological feature of non-asthmatic CRSwNP, with cell frequencies positively correlating with disease extent (41). Beyond this superantigen-mediated adaptive pathway, S. *aureus* cell wall components (including lipoteichoic acid and peptidoglycan) engage TLR2 and NOD2 on monocytes and macrophages, activating signaling cascades known to deposit H3K4me3 marks at pro-inflammatory gene promoters (26). In the CRS context, the persistent colonization of sinonasal mucosa by S. *aureus* biofilms represents a chronic epigenetic training stimulus, continuously reinforcing the activated chromatin state of mucosal macrophages (15, 35).

### 3.2 Fungal components: the β-glucan/dectin-1 trained immunity axis

Fungal sensitization, prevalent in type 2 CRSwNP and obligatory in allergic fungal rhinosinusitis, provides a second mechanistic connection to trained immunity. Fungal cell wall β-1,3-glucan engages Dectin-1 (CLEC7A) on macrophages and dendritic cells, activating Syk-CARD9-NF-κB signaling and inducing durable H3K4me3 deposition at pro-inflammatory gene loci (the canonical mechanism of β-glucan-induced trained immunity) (42). The seminal study by Quintin et al. demonstrated that monocytes trained by *Candida albicans*-derived β-glucan acquire enhanced responsiveness to secondary unrelated pathogens through this epigenetic mechanism (42). In the CRS mucosa, recurrent exposure to *Alternaria alternata*, *Aspergillus fumigatus*, and other aeroallergens that are major triggers of type 2 airway inflammation, may function as recurring epigenetic primers for sinonasal macrophages and ILC2s (24, 43). Beyond their allergenic protein components, fungal proteases disrupt epithelial tight junctions, amplifying alarmin release and further reinforcing the pro-inflammatory training milieu (15).

### 3.3 Viral respiratory infections and sinonasal epigenetic memory

Viral upper respiratory infections are major precipitants of CRS exacerbations and have been postulated to initiate mucosal trained immunity. Rhinovirus (RV) infection induces transcriptional and epigenetic changes in airway epithelial cells (affecting pathways related to mucosal immunity and barrier function) that persist well beyond viral clearance (44). Early-life RV infections are particularly relevant: murine studies demonstrate that neonatal rhinovirus infection establishes a lasting asthma-like phenotype dependent on IL-25, IL-33, TSLP, and IL-13-producing ILC2s, suggesting that early-life viral stimuli epigenetically train sinonasal ILC2s through alarmin-mediated mechanisms (45–47). Furthermore, BCG vaccination has been shown to protect against experimental viral infection in humans through the induction of cytokines associated with trained macrophage immunity, demonstrating the cross-protective potential of trained innate immune states against viral challenges (a paradigm directly applicable to understanding CRS-virus interactions) (27).

The SARS-CoV-2 pandemic has highlighted the capacity of respiratory viruses to induce lasting epigenetic reprogramming in monocytes and macrophages, with differential DNA methylation and histone modification patterns documented months after viral clearance (17, 21). Whether SARS-CoV-2-induced central trained immunity contributes to post-COVID olfactory dysfunction or CRS exacerbations (sequelae reported with elevated frequency in post-COVID populations) represents a clinically important hypothesis warranting systematic investigation.

### 3.4 Sinonasal dysbiosis: the microbiome as epigenetic modulator

The sinonasal microbiome is an underappreciated modulator of mucosal epigenetic homeostasis. Comprehensive single-cell profiling of sinonasal tissue from CRS patients by Wang et al. revealed distinct inflammatory cell signatures associated with specific microbial community compositions (44). The healthy sinonasal microbiome (dominated by commensals including *Lactobacillus* spp. and *Corynebacterium* spp.) maintains immune homeostasis partly through the production of short-chain fatty acids (SCFAs), which function as histone deacetylase (HDAC) inhibitors and promote tolerogenic epigenetic states in mucosal immune cells (48). In CRS, a dysbiotic state characterized by reduced microbial diversity and pathobiont enrichment depletes this endogenous SCFA-mediated epigenetic moderator, potentially enabling the unopposed establishment of pro-inflammatory trained immune states (44, 48). The exploitation of sinonasal microbiome restoration for epigenetic immunomodulation represents an emerging therapeutic frontier.

## 4 Epigenetic and metabolic reprogramming of mucosal macrophages in CRS

### 4.1 Inflammatory macrophage memory in CRS: clinical evidence

Tissue macrophages are among the most abundant innate immune cells in the sinonasal mucosa and represent a primary cellular substrate for trained immunity in CRS (6, 35). The pathological relevance of macrophage trained immunity to CRS has been most compellingly demonstrated by Haimerl et al., who integrated transcriptomics, genome-wide methylomics, and targeted metabolomics in monocyte-derived macrophages (MDMs) from patients with N-ERD (a severe CRSwNP endotype) and healthy controls (35). This study revealed that N-ERD MDMs exhibit a persistent pro-inflammatory phenotype characterized by an overall reduction in DNA methylation (indicative of an epigenetically open, transcriptionally primed state), aberrant metabolic profiles (including elevated intracellular acylcarnitines reflecting dysfunctional fatty acid oxidation) and markedly enhanced production of chemokines (CXCL1-3, CXCL8, CCL18, CCL20) and pro-inflammatory eicosanoids (5-LOX products, LTB4) upon secondary lipopolysaccharide stimulation compared to healthy and NSAID-tolerant CRS macrophages (35). Critically, these pro-inflammatory macrophage characteristics persisted following seven days of *in vitro* differentiation in the absence of any exogenous stimulation, demonstrating that the reprogramming is intrinsically epigenetically maintained.

Complementary evidence from lower airway disease further corroborates this concept. Lechner et al. demonstrated that macrophages from asthmatic patients acquire a TNF-dependent inflammatory memory, characterized by inflammatory transcriptional reprogramming and enhanced production of TNF-α, CCL17, leukotriene, and IL-6 upon restimulation with house dust mite (HDM) extract (49). This macrophage inflammatory memory was mediated through TNF signaling and was associated with epigenetic alterations consistent with trained immunity (49). Given the well-established shared immunological features between severe asthma and type 2 CRSwNP (with the two conditions co-existing in a large proportion of patients), these findings strongly implicate macrophage trained immunity as a conserved mechanism across the upper and lower type 2 airway inflammatory disease spectrum (24, 49).

### 4.2 Central trained immunity: bone marrow progenitor reprogramming in CRS

The persistence of trained macrophage states beyond individual cell lifespans demands an explanation at the hematopoietic progenitor level. Kaufmann et al. and Mitroulis et al. provided definitive evidence that epigenetic reprogramming of HSCs and myeloid progenitors in the bone marrow constitutes an integral component of systemic trained immunity, generating monocyte offspring with constitutively enhanced pro-inflammatory capacity (36, 37). In the CRS context, sustained systemic elevation of IL-1β, TNF, and IL-33 (all well-characterized inducers of central trained immunity) may epigenetically reprogram bone marrow progenitors to continuously supply pre-activated monocytes to the sinonasal mucosa (21, 37, 38). Furthermore, Koo et al. highlighted that epithelial-derived cytokines produced in excess by inflamed sinonasal epithelium (IL-25, IL-33, TSLP) directly affect HSC activation and differentiation, as well as basophil hematopoiesis, providing a mechanistic link between local mucosal inflammation and systemic innate immune reprogramming through the bone marrow (15). The therapeutic implications are significant: complete normalization of the sinonasal trained immune state may require intervention not only at the mucosal level but also at the hematopoietic progenitor compartment.

### 4.3 H3K4me3, H3K27ac, and the epigenetic landscape of CRS mucosa

At the molecular level, genome-wide epigenetic profiling of CRS tissue has begun to characterize the specific chromatin alterations that may underlie trained inflammatory states. Lal et al. conducted comprehensive genetic and epigenetic analyses of CRS tissue, identifying differentially methylated regions in genes involved in inflammatory signaling, cytokine production, and mucosal remodeling (48). Specific CpG sites in TSLP, IL-8, and PLAT promoters demonstrated significant hypomethylation in CRSwNP compared to controls, consistent with an epigenetically open, transcriptionally primed pro-inflammatory state (48). DNA methyltransferase inhibitors and HDAC inhibitors have been identified as potential pharmacological tools to reverse such epigenetic imprinting (48). The insight that these CRS-associated epigenetic changes overlap mechanistically with the histone modification patterns documented in trained macrophages (particularly H3K4me3 enrichment at pro-inflammatory gene promoters) provides a molecular bridge between the fields of CRS epigenetics and trained immunity (19, 48).

### 4.4 Metabolic reprogramming: the mTOR/HIF-1α/glycolytic axis in trained CRS macrophages

The metabolic underpinnings of macrophage trained immunity revolve around the mTOR/HIF-1α signaling axis and aerobic glycolysis (32, 34). mTORC1 activation in trained macrophages promotes HIF-1α stabilization, which transcriptionally upregulates glycolytic enzymes (driving the Warburg metabolic shift) (32). The resulting increase in acetyl-CoA production directly fuels histone acetylation (H3K27ac), while succinate accumulation inhibits prolyl hydroxylases and further stabilizes HIF-1α, thereby directly amplifying IL-1β production (34). In N-ERD macrophages, Haimerl et al. documented elevated acylcarnitines in macrophages, nasal lining fluid, sputum, and plasma (indicating a broad, multi-compartment dysregulation of immunometabolism), while genome-wide methylomics identified differential methylation at *CPT1A*, *CPT1B*, and *ACACA* loci (encoding key fatty acid oxidation enzymes), revealing the intersection of metabolic and epigenetic reprogramming in CRS macrophage training (35). This metabolic-epigenetic axis represents a tractable therapeutic target, as mTOR inhibitors could attenuate the glycolytic shift and downstream epigenetic enzyme activation that sustains the trained state (15, 32).

## 5 Trained ILC2s: innate lymphoid memory as a cellular amplifier of type 2 mucosal inflammation

### 5.1 ILC2 biology in CRS: from activation to training

ILC2s are tissue-resident, lineage-negative innate lymphocytes that lack rearranged antigen receptors and mount rapid type 2 cytokine responses (IL-5, IL-13, amphiregulin) upon activation by epithelial alarmins (43). ILC2s are markedly enriched in the nasal polyp tissue of CRSwNP patients, where their IL-5 and IL-13 production drives eosinophil recruitment, goblet cell metaplasia, and subepithelial fibrosis (key pathological hallmarks of type 2 CRS) (15, 43). The discovery that ILC2s can be epigenetically trained extends the trained immunity concept into the innate lymphoid compartment and provides a mechanistic basis for the non-antigen-specific amplification of type 2 inflammation observed in CRS (15, 50).

Martinez-Gonzalez et al. first demonstrated in murine lungs that allergen-experienced ILC2s acquire memory-like properties through unknown epigenetic mechanisms that remain incompletely characterized (50). These properties include their persistence in lymph nodes for 3–4 months after allergen clearance, elevated expression of IL-17RB (IL-25 receptor), and enhanced type 2 cytokine responses upon challenge with unrelated allergens. This seminal observation established that ILC2 memory is non-antigen-specific, non-adaptive, and dependent on initial IL-33 stimulation. Ebihara et al. subsequently provided a comprehensive synthesis of ILC2 training mechanisms, documenting the roles of alarmins, lipid mediators (PGD2 and cysteinyl leukotrienes), neuronal signals, and chromatin accessibility changes, including poised enhancers at effector cytokine gene loci, in driving the trained ILC2 phenotype (45). Importantly, this review also highlighted immunosuppressive IL-10-producing ILC2s as a counter-regulatory population induced by IL-33 and retinoic acid, and their expansion in CRSwNP nasal polyp tissue suggests that both pro- and anti-inflammatory ILC2 trained states coexist in CRS (45).

### 5.2 TLR4+ trained ILC2s in CRSwNP: a new cellular and molecular paradigm

Li et al. provided the first direct evidence for trained ILC2s in human nasal polyp tissue and delineated their molecular maintenance mechanisms with single-cell precision (51). Using scRNA-seq of sorted lineage-negative immune cells from five CRSwNP patients, Li et al. identified two ILC2 subsets: ILC2_c01, a quiescent naïve-like population, and ILC2_c02, characterized by elevated expression of memory cell markers (CD44, CCR8), type 2 cytokines (IL-5, IL-13), the alarmin receptor IL1RL1 (ST2), and TLR4 (51). Enrichment analysis revealed that pathways including IL-4/IL-13 signaling, toll-like receptor cascades, and MYD88-independent TLR4 signaling were significantly more active in ILC2_c02 than ILC2_c01. RNA velocity analysis suggested a developmental transition from ILC2_c01 toward ILC2_c02, with TLR4 and IL17RB expression increasing toward the trajectory terminus. The ILC2_c02/TLR4+ subset was specifically enriched in IL-5+ CRSwNP patients with high type 2 inflammation and positively correlated with tissue eosinophil counts, IL-13 expression (r=0.4471, P = 0.0049), and IL-5 levels (r=0.4801, P = 0.0023), establishing its clinical relevance.

Using Rag1−/− mice (lacking T and B cells) exposed to intranasal HDM challenge, Li et al. demonstrated that TLR4+ ILC2s persist in lung tissue during the four-week memory phase, while TLR4− populations contract (51). Upon secondary challenge with either HDM (related allergen) or papain (unrelated allergen), TLR4+ ILC2s mounted vigorous recall responses with IL-13 upregulation and enhanced mucus production, whereas TLR4− ILC2s did not, confirming their non-antigen-specific trained immunity properties. Critically, adoptive transfer of TLR4+ ILC2s (but not TLR4− ILC2s) into Rag2−/−Il2rg−/− mice produced significantly greater eosinophilia, IL-13 elevation, goblet cell hyperplasia, and airway hyperresponsiveness upon subsequent HDM challenge-establishing TLR4+ ILC2s as the key trained cellular effectors. Genetic ablation (*Tlr4−/−* mice) or pharmacological blockade (anti-TLR4 antibody) during the memory phase both significantly reduced IL-13+ ILC2 accumulation, mucus production, and inflammatory cell infiltration.

The molecular basis of TLR4+ ILC2 trained immunity was elucidated by scATAC-seq, which revealed increased chromatin accessibility in ILC2_c02 cells at the *Tlr4*, *Il1rl1*, *Gata3*, and *Il5* gene loci upon secondary allergen challenge (day 47 vs. day 45) (51). Transcription factor motif analysis identified AP-1 family members (JunB, Fos) and GATA3 as the key drivers of this chromatin accessibility, with JunB and JunD binding motifs contained within the *Tlr4* promoter region, and MafK (a downstream TLR4 effector) demonistrating elevated binding activity. These findings position AP-1-driven, TLR4-locus epigenetic reprogramming as a novel, lymphoid-specific mechanism of trained immunity, distinct from the Dectin-1/mTOR axis operative in trained macrophages.

### 5.3 Epigenetic mechanisms of ILC2 memory: chromatin poising and preparedness programs

Beyond the TLR4 locus, the epigenetic basis of ILC2 memory encompasses broad chromatin remodeling at effector gene regulatory elements (52). Verma et al. demonstrated in a murine asthma model that trained ILC2s establish a specific epigenetic landscape characterized by increased ATAC-seq accessibility at loci encoding IL-5, IL-13, and key transcription factors. Much like the epigenetic changes that distinguish memory from naïve CD8+ T cells, this landscape allows repetitive allergenic stress to activate a “preparedness program”, enabling rapid and amplified secondary responses (52). These chromatin accessibility patterns, established by initial allergen or IL-33 exposure and maintained through cell division, encode an epigenetic “inflammatory memory” that is independent of ongoing antigen stimulation. Steer et al. further demonstrated that lung ILC2s are trained by endogenous neonatal IL-33, acquiring long-lasting enhanced inflammatory responsiveness that persists into adulthood, implicating early-life sinonasal microbial exposures in establishing ILC2 trained immunity relevant to adult CRS (47).

### 5.4 Regulatory counterbalance: IL-10-producing trained ILC2s

The trained ILC2 compartment is not uniformly pro-inflammatory. Under conditions of severe or prolonged allergen exposure, IL-33 combined with retinoic acid, IL-2, or IL-27 induces immunosuppressive IL-10-producing ILC2s, which are characterized by the upregulation of exhaustion markers such as PD-1 and TIGIT, alongside a diminished production of the effector cytokines IL-5 and IL-13 (45). An increase in IL-10-producing ILC2s in nasal polyp tissue from CRSwNP patients has been documented, potentially representing a homeostatic counter-regulatory response to uncontrolled type 2 inflammation. Furthermore, increased IL-10-producing ILC2s in peripheral blood have been associated with improvement in allergic rhinitis during allergen-specific immunotherapy (45). Whether therapeutic manipulation of this immunosuppressive trained ILC2 population (for example, through targeted retinoic acid delivery to the sinonasal mucosa) represents a viable immunomodulatory strategy for CRS warrants investigation.

## 6 Epithelial stem cell memory: the non-immune frontier

### 6.1 Nasal epithelial cells as active architects of mucosal immunity

The sinonasal epithelium is recognized as an active immunological organ rather than a passive physical barrier (53, 54). Nasal epithelial cells sense inhaled pathogens and allergens through pattern-recognition receptors, produce the canonical type 2-driving alarmins (IL-25, IL-33, TSLP), recruit and activate dendritic cells, and directly modulate T cell differentiation (15, 53). The nasal epithelium encompasses a hierarchical cellular architecture (including basal progenitor cells, ciliated columnar cells, goblet cells, club cells, and rare ionocytes) as revealed by scRNA-seq (55). Basal cells serve as multipotent stem cells responsible for maintaining epithelial homeostasis and replenishing differentiated populations in response to injury (46, 53, 55, 55). In CRS, conspicuous hyperplasia of undifferentiated basal cells coexists with impaired differentiation into ciliated and secretory cells, a pattern directly driven by type 2 cytokine exposure through insulin receptor substrate (IRS)-dependent signaling (15).

### 6.2 Allergic inflammatory memory in nasal epithelial progenitors

The concept of epithelial stem cell trained immunity in the airway received transformative validation from Ordovas-Montanes et al., published in *Nature* in 2018 (56). Using chromatin accessibility profiling (ATAC-seq) of human nasal epithelial progenitor cells from atopic individuals and healthy controls, and complementary *in vitro* experiments, this study demonstrated that nasal basal cells from individuals with allergic inflammation maintain heritable chromatin accessibility profiles at inflammatory gene loci, including regulatory elements of TSLP and IL-33; such profiles are preserved *in vitro* long after removal from the inflamed tissue environment. These epigenetically reprogrammed basal cells exhibited constitutively enhanced alarmin production capacity upon secondary cytokine stimulation, demonstrating that the inflammatory memory is encoded in and perpetuated through the epithelial progenitor compartment.

In the CRS context, Wang et al. directly demonstrated that type 2 cytokines (IL-4 and IL-13) impair basal cell differentiation through IRS-dependent signaling, contributing to loss of ciliated and secretory cells in CRSwNP (57). Crucially, Koo et al. synthesized evidence demonstrating that basal cells from CRSwNP patients acquire inflammatory memory following IL-4 and IL-13 exposure, thereby establishing epithelial stem cell trained immunity as a CRS-relevant phenomenon (15). These epigenetically imprinted basal cells, residing in the sinonasal tissue after ESS and biologic therapy, represent a potential reservoir of pro-inflammatory memory capable of re-initiating inflammatory cascades upon renewed environmental stimulation, thus providing a mechanistic basis for post-surgical CRS recurrence (57).

### 6.3 Inflammatory memory across epithelial tissues: universal principles

The paradigm of epigenetic inflammatory memory in epithelial stem cells has been validated across multiple tissues. Naik et al. demonstrated in a murine psoriasis model that skin epithelial stem cells retain altered chromatin accessibility at inflammatory gene promoters following resolution, sensitizing them to mount amplified inflammatory responses upon secondary challenge (58). The principles of this skin inflammatory memory are broadly conserved: Francis et al. documented analogous inflammatory chromatin imprinting in psoriatic skin even during clinical remission, providing molecular insight into the well-recognized recurrent nature of psoriatic disease (59). The parallel with CRS, where clinical remission achieved by ESS or biologics does not eliminate mucosal epigenetic inflammatory memory, is conceptually compelling. More broadly, Naik and Fuchs articulated the concept of “inflammatory memory and tissue adaptation”, in which repeated inflammatory insults progressively reshape the epigenetic landscape of epithelial stem cells, shifting their inflammatory set-point (60). In CRS, where patients experience repeated cycles of infection, allergen exposure, and immune activation over years, this progressive epigenetic ratcheting may constitute a fundamental mechanism of disease chronification (60).

## 7 Therapeutic implications: targeting the sinonasal epigenetic inflammatory landscape

**Figure 3:**
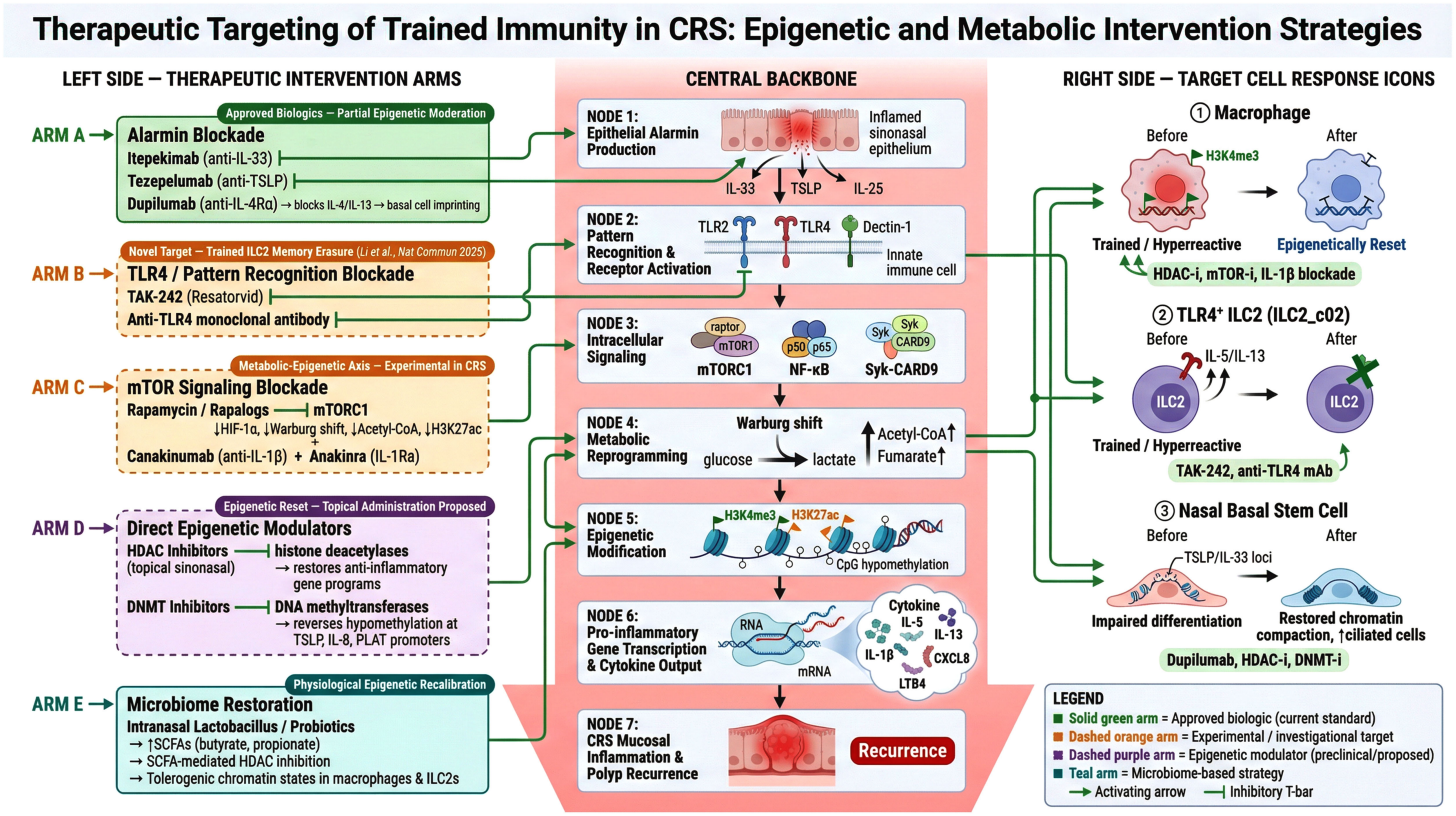
Therapeutic targeting of trained immunity in CRS: epigenetic and metabolic intervention strategies. A central seven-node backbone depicts sequential trained immunity induction, spanning from epithelial alarmin production (Node 1) and pattern recognition (Node 2) through intracellular signaling (Node 3), metabolic reprogramming (Node 4), epigenetic modification (Node 5), and pro-inflammatory cytokine output (Node 6), culminating in polyp recurrence (Node 7). Five intervention arms target discrete nodes. Arm A (itepekimab, tezepelumab, dupilumab) intercepts upstream alarmin signals with partial epigenetic moderation. Arm B (TAK-242, anti-TLR4 mAb) targets TLR4, the key trained ILC2 receptor. Arm C (rapamycin, canakinumab, anakinra) disrupts the mTOR/HIF-1α metabolic-epigenetic axis. Arm D (topical HDAC-i and DNMT-i) directly reverses pathological histone marks and promoter hypomethylation. Arm E (intranasal Lactobacillus/probiotics) leverages SCFA-mediated HDAC inhibition for physiological epigenetic recalibration. Right-panel schematics show predicted outcomes: macrophage epigenetic reset, TLR4^+^ ILC2 memory erasure, and restoration of nasal basal cell differentiation. CRS, Chronic Rhinosinusitis; DNMT-i, DNA Methyltransferase Inhibitor; HDAC-i, Histone Deacetylase Inhibitor; HIF-1α, Hypoxia-Inducible Factor 1-alpha; ILC2, Group 2 Innate Lymphoid Cell; mAb, Monoclonal Antibody; mTOR, Mechanistic Target of Rapamycin; SCFA, Short-Chain Fatty Acid; TLR4, Toll-Like Receptor 4.

Therapeutic targeting of trained immunity in CRS was displayed in **Figure 3**.

### 7.1 Current biologics through the trained immunity lens

The clinical efficacy of dupilumab and other type 2 targeted biologics in CRSwNP is now well established (10, 13). However, the near-universal disease relapse following biologic discontinuation reveals the inability of these agents to erase the underlying epigenetic inflammatory memory (10, 15). Bangert et al. demonstrated that pathogenic memory Th2 cells, mature dendritic cells, and Tc2 cells persist in the skin of atopic dermatitis patients following clinical resolution under dupilumab, thereby underscoring that cytokine blockade attenuates but does not eliminate epigenetic cellular memory (61).

Through the trained immunity framework, the molecular mechanisms and limitations of current biologics can be newly understood. Dupilumab, by blocking IL-4 and IL-13 signaling, likely suppresses type 2 cytokine-induced epigenetic imprinting of nasal basal cells, thereby functioning as a partial epithelial epigenetic moderator. Itepekimab (anti-IL-33) and tezepelumab (anti-TSLP), targeting upstream epithelial alarmins, may interrupt both peripheral and central induction of trained immunity by the inflamed epithelium (15, 62). However, none of these agents directly address the epigenetic modifications already deposited in trained macrophages, TLR4+ ILC2s, or epigenetically imprinted basal cells, explaining the reversibility of their clinical effects (61).

### 7.2 Epigenetic modulators: HDAC inhibitors and DNA methylation agents

The reversible nature of histone modifications, which constitutes the mechanistic foundation of trained immunity, offers direct therapeutic leverage through epigenetic modulators. HDAC inhibitors, by promoting histone acetylation and reactivating anti-inflammatory gene programs, may reset maladaptive trained immunity in sinonasal macrophages and ILC2s (48). DNMT inhibitors may restore the expression of epigenetically silenced anti-inflammatory genes through reversal of aberrant hypermethylation, thereby complementing strategies targeting pro-inflammatory chromatin states to collectively restore epigenetic homeostasis. The topical administration of these agents to the sinonasal mucosa via nasal irrigation or nebulization, thereby bypassing the systemic toxicity concerns that have constrained their oncological use, represents a potentially feasible translational approach requiring prospective investigation (13).

### 7.3 Targeting the mTOR/metabolic axis and IL-1β signaling

Given the central role of mTOR activation in driving trained immunity-associated metabolic reprogramming, mTOR inhibitors (rapamycin and analogs) emerge as potential agents for suppressing maladaptive trained immunity in CRS macrophages (15). mTOR inhibition is predicted to attenuate the Warburg metabolic shift, reduce fumarate accumulation, and diminish the epigenetic histone demethylase inhibition that sustains H3K4me3 marks at pro-inflammatory loci (32). For non-type 2 CRS, where IL-1β-producing macrophages are pathogenic hallmarks, IL-1β blockade with canakinumab or IL-1R antagonism with anakinra, when combined with mTOR inhibition, may synergistically abrogate macrophage trained immunity (15).

### 7.4 Anti-TLR4 strategies: directly targeting trained ILC2 memory

The identification of TLR4 as a critical regulator and surface marker of trained ILC2s in CRSwNP tissue opens a novel therapeutic avenue (51). Li et al. demonstrated that both genetic TLR4 ablation (*Tlr4−/−* mice) and pharmacological TLR4 blockade (anti-TLR4 monoclonal antibody) significantly reduced IL-13+ ILC2 accumulation, mucus production, tissue eosinophilia, and airway hyperresponsiveness *in vivo* (51). The TLR4 antagonist TAK-242 (resatorvid), which inhibits TLR4 signaling by binding to TLR4’s intracellular domain, was shown to reduce IL-13 production in *in vitro*-trained ILC2s, demonstrating pharmacological tractability. Local administration of anti-TLR4 strategies to the sinonasal compartment as an adjunct to ESS or biologic therapy represents a mechanistically grounded hypothesis meriting clinical investigation (63).

### 7.5 Microbiome restoration and probiotic immunomodulation

Restoration of the dysbiotic sinonasal microbiome to a composition enriched in SCFA-producing commensals represents a physiologically motivated, potentially low-risk strategy for epigenetically recalibrating sinonasal trained immunity (44). SCFA-mediated HDAC inhibition promotes tolerogenic epigenetic states in mucosal macrophages and ILC2s, counteracting the pro-inflammatory chromatin landscapes established by pathobiont-mediated training stimuli. The Lal et al. review highlights the interaction of microbial metabolites with epigenetic regulation in CRS (48). Clinical studies of intranasal *Lactobacillus* preparations have demonstrated preliminary signals of symptom improvement in CRS (44). Future mechanistic studies should determine whether microbiome interventions produce measurable epigenetic changes, including H3K4me3 and H3K27ac profiles, in sinonasal innate immune and epithelial cells.

## 8 Future research directions

The trained immunity framework in CRS generates a rich and clinically actionable research agenda. At the mechanistic level, comprehensive single-cell multi-omic profiling of CRS sinonasal tissue, integrating scRNA-seq, scATAC-seq, and spatial transcriptomics, will be essential to map the full epigenetic landscape of trained macrophages, ILC2 subsets, and epithelial progenitors across CRS endotypes and disease stages (48, 51). The discovery of TLR4+ trained ILC2s in nasal polyp tissue exemplifies the transformative discovery potential of such approaches and strongly suggests that additional trained innate immune subsets, potentially including trained mucosal dendritic cells, mast cells, and ILC1s, remain to be characterized in CRS (48, 51).

Translational research should focus on validating epigenetic biomarkers of trained immunity as predictors of post-surgical CRS recurrence. Candidate biomarkers include H3K4me3 enrichment scores at pro-inflammatory gene promoters in tissue macrophages or circulating monocytes, DNA methylation profiles at CRS-associated CpG sites in nasal brushing specimens, and acylcarnitine signatures in nasal lavage fluid or plasma (48). Such biomarker-guided risk stratification could enable the rational selection of patients who would benefit most from adjunctive epigenetic therapies following standard CRS treatment.

A critical open question concerns whether currently approved biologic therapies exert disease-modifying epigenetic effects with long-term treatment. Prospective studies incorporating longitudinal nasal epithelial biopsy sampling with chromatin accessibility profiling before, during, and after biologic therapy, as well as at defined intervals following discontinuation, are needed to determine whether biologics reduce the ‘inflammatory epigenetic set-point’ of nasal progenitors and innate immune cells, and whether such epigenetic effects correlate with sustained remission (64). The development of organoid models incorporating innate immune-epithelial interactions would further facilitate the *in vitro* testing of epigenetic therapeutic strategies before clinical implementation (57).

## 9 Conclusion

CRS recurrence and therapy resistance represent unresolved clinical imperatives that demand mechanistic frameworks beyond conventional adaptive immunity models. This review has presented trained immunity, the epigenetic and metabolic reprogramming of innate immune and epithelial cells, as a fundamental and underappreciated driver of CRS chronification. The sinonasal mucosa is continuously exposed to the key inducers of trained immunity: *S. aureus* biofilms, fungal β-glucans, respiratory viruses, and dysbiotic microbial communities, each of which epigenetically imprints mucosal macrophages, ILC2s, and basal progenitor cells with heritable pro-inflammatory states. The recent single-cell characterization of TLR4+ trained ILC2s in human nasal polyp tissue, maintained by AP-1/JunB-driven chromatin remodeling at the *Tlr4* locus, exemplifies the cellular and molecular specificity of this phenomenon and identifies a novel therapeutic target. Concurrently, nasal basal stem cells encode inflammatory epigenetic memory through cytokine-induced chromatin accessibility changes that perpetuate mucosal immune reactivity independent of ongoing stimulation.

The trained immunity framework fundamentally reconceptualizes CRS as a condition sustained by epigenetically encoded mucosal inflammatory memory, rather than by persistent antigen stimulation alone, with correspondingly far-reaching implications for therapeutic strategy. Targeting the epigenetic and metabolic pathways of trained immunity through HDAC inhibitors, mTOR inhibitors, anti-TLR4 strategies, and microbiome restoration provides a mechanistic approach for disease modification. Future clinical translation will require integrating single-cell epigenomics, biomarker identification, and targeted sinonasal drug delivery.
